# Research on the “Privacy Paradox” behaviors of online users from the perspective of attitude ambivalence: ERPs evidence

**DOI:** 10.1371/journal.pone.0330438

**Published:** 2025-08-20

**Authors:** Qiuhua Zhu, Rui Sun, Dong Lv, Yiyang Shen, Shukun Qin

**Affiliations:** 1 Chen Shouren Business School, Quanzhou Normal University, Fujian, China; 2 School of Business Administration, Huaqiao University, Quanzhou, Fujian, China; University of Education, PAKISTAN

## Abstract

The “privacy paradox” phenomenon and its explanations are becoming impediments to the correct identification of user privacy attitudes, and understanding the link between privacy attitude instability and behavior helps enterprises to correctly interpret user privacy needs, ensuring the long-term and steady growth of digital businesses. Grounded in the bounded theory of limited cognitive resources and associative-propositional evaluation model (APE model), this research employs ERP technology, targeting the instability of individual attitudes, the study introduces the usefulness of recommendation information and the intrusiveness of recommendation information, privacy intrusiveness and information interestingness, recommendation usefulness and information aversion as experimental stimulus variables of attitude ambivalence, and explores the internal cognitive mechanism of user privacy attitude ambivalence and disclosure behavior. The results show: (1) Under conditions of high intrusiveness of recommendation information, users are more prone to disclose privacy if the recommendation is viewed as highly useful compared to when it is less useful; there is no noteworthy disparity in privacy disclosure under conditions of low informational intrusiveness. (2) Relative to conditions of both high informational intrusiveness with low usefulness, and low privacy intrusiveness combined with low information interestingness, users manifest a higher propensity for privacy disclosure under the circumstances of low privacy invasiveness with highly information interestingness, and high recommendation usefulness along with high information aversion, prompting reduced amplitudes in P2 and N2 waves. Moreover, a larger P2 wave amplitude is evoked when there is lower perceived usefulness in recommendations coupled with less information aversion, as contrasted with higher perceived usefulness and higher aversion. This study examines the cognitive mechanism between privacy attitude ambivalence and disclosure conduct, presenting a fresh perspective for research on the “privacy paradox”, enabling businesses to deeply grasp the instability of user attitudes in conjunction with behavioral responses, significantly contributing to better protection of user privacy and the improvement of the user experience.

## 1. Introduction

In the era of digital intelligence, “panning for gold” from user data is becoming the core of enterprise value creation [1]. User information constitutes the raw material necessary for firms to craft digital products, utilizing the privacy data disclosed by user, businesses employ intelligent algorithms to excessively “probe” into varying consumer demands, and thereby redefining their product positioning, service and profit model, in order to deliver targeted products and services to users, enabling companies to achieve a “behavioral surplus.” However, in the wave of digitalization, while user privacy data empowers industries and improves economic benefits, it leads to the users’ interests preferences and dynamic information being under perpetual “intelligent surveillance” and “digital gaze” [2]. Companies deploy intelligent algorithms to entice users into revealing personal details in trade for complimentary services, or amidst online transactions, persuading users to compromise their privacy for the sake of conveniences, embodying the privacy paradox [2,3]. The privacy paradox exemplifies the ambivalent attitude users hold towards privacy, as they pursue convenience and personalized services while remaining apprehensive about privacy violations and data misuse. This paradox underscores the psychological conflicts underlying user behavior and poses challenges to the long-term sustainability of businesses, eliciting repercussions such as user attrition and credibility erosion. For instance, Facebook achieves precise advertising placement by analyzing user behavior, interests, and social networks [4]; however, its frequent privacy breaches have triggered significant concerns among users regarding data security [5]. While enjoying free services, users experience a sharp decline in their trust toward the platform [6], leading them to take actions such as reducing information sharing or uninstalling applications to protect their privacy. This reaction reflects the conflicting psychology of users between convenience and privacy protection. Event-related potentials (ERPs) technology enables an in-depth exploration of this psychological contradiction and its effects on privacy disclosure behavior. By continuously monitoring changes in users’ psychological states during decision-making, ERPs can uncover users’ emotional reactions and cognitive processes when facing privacy risks. This technology not only assists researchers in understanding users’ attitudes and behavioral patterns regarding privacy protection but also provides essential practical guidance for businesses, enabling them to develop more effective privacy protection strategies. Consequently, understanding this psychological conflict and its effects on privacy disclosure is vital for achieving a balance between corporate profitability and user privacy.

Research has found that despite high levels of privacy concern, users will still choose to disclose personal information on many occasions, a phenomenon called the privacy paradox [7]. Early studies often used self-reported research designs to explore the trade-offs between risk and reward in users’ privacy decisions [6,8], which relied primarily on users’ subjective judgments forming generalized evaluations, hypothetical responses to assumed scenarios, which do not correspond with real online contexts. Current research often makes use of more sophisticated technologies such as magnetic resonance imaging (fMRI) and event-related potentials (ERP), overcoming the past limitations of unreal and unspecific measurements of privacy attitudes, better matching the cognitive processing traits of user decision-making in real scenarios [9]. Some researches leverage fMRI technology [10], to locate the activation of specific functional brain areas during the decision-making process [11,12], informed by the theory of privacy calculus, thereby explaining the reasons for the emergence of the privacy paradox; Another set of studies utilize ERP technology [13], by identifying its characteristics in cognitive processing time, presenting specific brain electrical signals within a matter of milliseconds, based on self-perception theory, and attitude metacognitive theory, to explain the privacy paradox from the perspectives of attitude formation and stability [14,15]. Although these literatures systematically interpret the ‘ privacy paradox ‘ from multiple perspectives, an implicit assumption of current research is that privacy attitudes are stable. This assumption does not necessarily hold when users make real decisions. It ignores the unstable characteristics of users ‘ privacy attitudes in real situations. In particular, when users weigh privacy risks and benefits, positive and negative views coexist, resulting in users ‘ more contradictory privacy decisions. However, the existing research lacks the exploration of the conflict and contradictory views of the user ‘s attitude system itself. The ambivalence of user ‘s privacy attitude may be the key factor leading to the ‘ privacy paradox ‘.

Building on this, the research takes the instability of individuals’ attitudes as the subject of its inquiry, exploring the effect of users’ attitude ambivalence on privacy disclosure behavior, and further probes the internal mechanism of action of their own cognitive resources on privacy disclosure under inconsistency between users’ cognition and emotions. The study design introduces the perceived usefulness and intrusiveness of recommendation information to induce attitudinal ambivalence in individuals, combining event-related potential experimental technology (ERPs), to amass neuroelectric data pertaining to the privacy decision-making process. Investigating the impact of ERPs technology and attitude ambivalence on user privacy behavior is crucial, primarily due to ERPs unique high temporal resolution and its ability to directly measure cognitive processes. Privacy disclosure is frequently influenced by intricate emotional and cognitive factors, yet traditional self-report methods often fail to effectively capture users’ immediate psychological states when confronted with privacy risks, as these methods are susceptible to social desirability biases and memory distortions. ERPs technology enables real-time recording of individual brain activity, providing physiological data on users’ cognitive responses during privacy-related decision-making processes, thereby more accurately reflecting the ambivalent attitudes formed in real-time as individuals weigh risks and benefits [14,15]. For instance, we can observe how specific ERPs components (such as P2 and N2) mirror users’ emotional arousal and cognitive conflict when processing privacy-related information. These physiological indicators offer insights into the subconscious psychological and cognitive “black box” of users’ instantaneous ambivalence between privacy risks and benefits. Thus, it furnishes a more precise elucidation of the discrepancies between attitudes and behaviors.

ERPs technology shows remarkable advantages in researching online user privacy behavior, especially in comparison with other neuroimaging methods. In complex contexts such as high-risk and low-risk information sharing, ERPs can promptly and authentically capture the subtle differences in users’ cognitive load and emotional reactions, offering profound insights into privacy behavior. In contrast to conventional subjective measurement techniques, ERPs technology effectively compensates for data bias, offering a more scientific foundation for the development of privacy policies. In comparison with functional magnetic resonance imaging (fMRI), the strengths of ERPs technology include its real-time performance and ecological validity. While fMRI can uncover the effects of cognitive emotions on privacy behavior, it faces limitations related to environmental conditions, mobility, and high costs, alongside low temporal resolution. Conversely, ERPs technology not only simplifies experimental control but also captures real-time changes in cognition and emotion within genuine online decision-making contexts, effectively exposing users’ conflicted mental states in privacy situations. Such immediate feedback capacity allows researchers to better understand how online users react to privacy behavior in dynamic contexts, thereby providing more specific suggestions for policy development. Consequently, this research utilizes ERPs technology to conduct a deeper analysis of how users’ immediate unstable attitudes (the inconsistency in attitude ambivalence and the structure of cognitive and emotional factors) affect privacy disclosure behavior, uncovering the specific psychological mechanisms at play during the decision-making process, thereby expanding the application of the limited cognitive resources theory and the APE model in the field of user privacy disclosure behavior.

## 2. Research status and theoretical basis

### 2.1. Research status of privacy paradox

The “privacy paradox” originatied in the medical field, with researchers discovering that patients did not want their privacy information disclosed, yet an authoritative explanation from the researchers would often change the patients’ attitudes [16]. Subsequently, research gradually extended to the field of online consumption. In one study on online shopping, researchers identified the “privacy paradox” by measuring users’ privacy attitudes and observing how online shoppers process privacy data. Even though individuals articulate concerns regarding privacy invasions, when benefits are presented, they are still prepared to surrender personal information to online sellers [17].

Initial studies on the “privacy paradox” mainly focused on a few aspects: First, they were premised on the “rational actor” model, creating frameworks such as the Privacy Calculus theory, theorizing that the reasons for the inconsistency between user privacy attitudes and behavior is because the benefits of disclosing privacy are greater than the risks, and despite concerns about disclosing privacy, individuals still choose to disclose personal information [7,18]. In truth, humans are irrational, subjected to a host of biases and deviations. Therefore, building on the first category, the assumption of the “irrational individual” was introduced, constructing a comprehensive explanatory framework such as the Privacy Calculus – Explanation Level Integration Framework, theorizing that various cognitive biases present in individuals, such as control illusions [6,19], demands for immediate satisfaction, and optimistic bias [20,21], lead to users overestimating current benefits and underestimating future risks, leading them to ultimately disclose their privacy. Thirdly, the notion that individuals’ actions regarding privacy disclosure echo the effects found in quantum measurement—outcomes are only ascertained when decision-making culminates—gave rise to what’s termed the “quantum theory” [22].

Current studies employing more refined technologies like magnetic resonance imaging (fMRI) and event-related potentials (ERP) have overcome past limitations of unrealistic and non-specific measurements of privacy attitudes, aligning more closely with the cognitive processing characteristics of user brains during decision-making in realistic situations [9]. Specifically, a segment of research makes use of fMRI techniques [10], have identified the activations in brain areas such as the caudate nucleus and the medial prefrontal cortex, along with the amygdala and the insula during the decision-making processes [11,12], grounding their findings in Privacy Calculus Theory, finding that the activation of specific brain areas correlates positively with perceived benefits and risks, thus attempting to explain the causes behind the privacy paradox; while other research harnessing event-related potential technology [13], sought to identify cognitive processing characteristics within specific timing, revealing precise individual brain electrical signals within a scope of a few hundred milliseconds, on the basis of Self-Perception Theory and Attitude Metacognition Theory, have explored the privacy paradox from the angles of both the formation of privacy attitudes and the true, specific, and deep metacognitive attitudes [14,15].

In summary, early studies predominantly used self-report research designs to examine the trade-offs between risks and benefits that users consider when making privacy decisions [6,8], primarily based on the users’ subjective judgments, leading to general assessments that are hypothetical responses to hypothetical scenarios, not aligning with real-life online contexts. Existing research taking advantage of magnetic resonance imaging and event-related potential technologies [10,11] allows for more detailed and authentic inquiries which overcome previous unrealistic measurement limitations on privacy attitudes [9]. Despite these studies carrying out a systematic investigation of the “privacy paradox,” an implicit assumption of current research is the stability of privacy attitudes, a presumption that may not consistently hold true, as the attitude system includes both stability and instability, overlooking the characteristics of instability in users’ attitude systems in real contexts, with users harboring both positive and negative views when evaluating privacy risks and benefits, resulting in increased ambivalence during their privacy decisions, and there is a lack of exploration into the conflicts and contradictory viewpoints within users’ attitude systems themselves in existing research. Given this, our study utilizes event-related potential technology (ERPs). This method has a high temporal resolution, allowing for the timely capture of physiological electrical signal changes in users within hundreds of milliseconds, thus more accurately reflecting the unstable attitudes that individuals construct in real time. While fMRI can measure the influence of cognitive and emotional factors on privacy behavior, it faces challenges related to environmental restrictions, high expenses, and low temporal resolution. ERPs technology allows for the analysis of specific psychological phenomena regarding privacy disclosure by examining the inconsistencies in users’ cognitive and emotional states through objective biological electrical signal measures, focusing on individual attitude instability, to explore the impact of users’ attitudinal ambivalence on privacy disclosure behavior, and further delves into the inner workings of how personal cognitive resources act upon privacy disclosure under inconsistencies between cognition and emotion. The study marries the theory of limited cognition and the APE model, aspiring to proffer a novel interpretative framework for the “privacy paradox.”

### 2.2. Theoretical basis

#### 2.2.1. Theory of limited cognitive resources.

Based on information processing theory, filtering, controlling, retaining, and processing information all require cognitive resources, and the limitation of cognitive resources directly determines the complexity and quantity of information that an individual can process [23]. The concept of limited cognitive resources was initially introduced by Sweller et al. in 1988, highlighting that individuals need to invest “mental effort” to accomplish tasks, which relies on specific cognitive resources during information processing [24]. Completing each task consumes specific cognitive resources, and when performing multiple tasks simultaneously, these resources can be partially shared. However, an individual’s total cognitive resources are limited, making resource allocation crucial when facing multiple tasks. According to cognitive resource theory, individuals consume corresponding cognitive resources when identifying and processing tasks. The complexity of a task directly affects the amount of cognitive resources required; complex tasks often demand more cognitive resources, which can lead to rapid depletion of an individual’s cognitive resources. Specifically, when task complexity exceeds an individual’s processing capacity, information overload may occur, adversely affecting the efficiency and quality of task execution. For example, in the context of online shopping, research has examined the impact of recommendation systems on users’ willingness to reuse, finding that product presentation and the complexity of the website pages significantly affect users’ emotional and cognitive processes. When page complexity is excessively high, users face overload in emotional and cognitive capacities, resulting in negative emotions and consequently reducing user satisfaction and willingness to reuse [25]. This empirical study further validates the significance of the limited cognitive resources theory in practical applications, emphasizing that maintaining a moderate level of complexity is crucial for supporting users’ cognitive processing when designing information systems.

#### 2.2.2. Associative-propositional evaluation model.

The Associative-Propositional Evaluation Model (APE) constructs the concept of attitude based on two distinct cognitive psychological processes: associative processing and propositional processing, providing an important perspective for understanding the limitation of cognitive resources during information processing [26]. Associative processing is defined as the activation of associations in memory, primarily relying on the interaction between external stimuli and memory characteristics. This process is automatic, and individuals’ emotional responses to specific stimuli are largely driven by prior cognitive structures, independent of truth judgments and subjective correctness [26,27]. In contrast, propositional processing entails the activation of information suggested by the activated associations; this process is more logical and reasoning-oriented, depending on truth values and closely tied to subjective judgments [28]. During propositional processing, the automatic emotional responses resulting from associative processing are incorporated into the thinking system, resulting in corresponding propositions [29]. Propositional processing evidently consumes more cognitive resources because it encompasses logical reasoning and intricate judgment processes. In the course of information processing, the finite nature of an individual’s cognitive resources suggests that encountering complex or multiple information may result in cognitive overload. The APE model highlights the interplay between associative and propositional processing, encompassing how associations influence propositions, propositions affect associations, and how both jointly shape behavior [30]. For instance, when individuals face information overload in complex contexts, automatic associative processing may take up a significant amount of cognitive resources, thus impacting the effectiveness of propositional processing and leading to a lack of adequate resources for thoughtful deliberation during judgment formation. Thus, comprehending the associative and propositional processing in the APE model aids in clarifying the role of the limited cognitive resources theory in attitude formation and change. When processing information, individuals require a more judicious allocation of their limited cognitive resources in increasingly complex situations and tasks to effectively evaluate attitudes and make decisions regarding behaviors.

## 3. Research hypotheses

In social psychology, contradiction is seen as a type of unconscious conflict inherent within an individual’s essence. Research has indicated that attitudinal ambivalence arises when an individual harbors concurrent positive and negative affective responses towards a target object [31]. Scholars have further noted that attitudinal ambivalence is when an individual has both positive and negative evaluations towards a target object simultaneously [32]. Despite variations in scholars’ interpretations of attitudinal ambivalence, it is generally agreed that it manifests when individuals have both positive and negative evaluations toward the same entity, indicative of a weaker form of attitude strength and is characterized by instability. Attitudinal ambivalence can be generated by external stimuli such as cognitive needs, value conflicts, and emotional conflicts.

In smart recommendation scenarios, companies often subtly persuade users to disclose their personal data by the usefulness of the recommended information; however, as the precision of smart recommendations improves, the invasion of user’s information becomes increasingly evident. On one hand, users are concerned about the infringement of their privacy by the recommended information, on the other hand, they are trapped by the “digital dividend” and cannot extricate themselves. Consequently, this study selects the usefulness and the intrusiveness of recommendation information as situational cues driving attitudinal ambivalence. According to the tri-component model of attitudes that includes cognition, affect, and behavior, attitudinal ambivalence encompasses both cognitive elements and affective components of conflict and contradiction. Given that both usefulness and invasiveness of recommended information pertain to cognitive and emotional elements, in order to profoundly dissect the internal cognitive mechanics of user attitudinal ambivalence toward privacy disclosure, this study further categorizes the usefulness of recommended information into usefulness and interestingness through cognitive and emotional factors. The intrusiveness of recommended information segmented into privacy intrusiveness and information aversion, setting the stage to investigate the intrinsic cognitive sequences underpinning individual behavioral choices within the dichotomy of cognitive and emotional inconsistencies.

### 3.1. Behavioural hypotheses

Usefulness refers to the degree to which an individual subjectively believes that using a specific technology or product can improve their work performance and efficiency [33]. The usefulness of recommended information is about users disclosing personal information to digital businesses with the expectation of improved work efficiency. Research indicates that the usefulness of recommended information can elevate online users’ recognition of its functional capacity and perception of emotive requirements. Some research has uncovered that the usefulness of recommendation information is capable of supplying tailored products and services to users, which consequently boosts their trust, contentment, and enduring usage intentions, exerting a positive effect on their predisposition to disclose personal information [34]. Furthermore, the pertinence of recommended content on social networks can address an assortment of affective necessities for users, offering enhanced sensations of social integration, interpersonal requisites, social acknowledgement, and enjoyment. Wang and colleagues’ study demonstrates that perceived usefulness positively influences user intentions to disclose personal information [35]. Stone and others found a significant positive correlation between usefulness and the willingness to disclose. When users believe that disclosing their information can lead to more interpersonal relationships and a sense of belonging, their willingness to disclose personal privacy is stronger, meaning that the greater the perceived usefulness, the more proactive the disclosure intention [36].

The intrusiveness of recommendation information denotes the subjective negative perception users have when platforms tailor recommendations too closely from sensitive user information, making them feel their privacy is being breached [37]. Smart recommendations derived from users’ sensitive information can augment the perceived threat to privacy and strengthen anxieties regarding privacy compromise risk [38], thus adversely affecting inclination to share private data [39]. Privacy intrusion by personalized recommendations significantly escalates user’s concerns over privacy; the more pronounced these worries, the more resistant users become to privacy disclosure [40,41]. The loss of digital rights due to the intrusiveness of recommendation tailoring induces negative perceptions such as vigilance and panic among users. Research demonstrates that the intrusiveness of recommendation information can negatively affect the willingness to disclose privacy [42] and adversely affect the effectiveness of privacy policies, with intrusiveness bolstering the user’s motivation for privacy protection, thereby engendering distrust in privacy measures formulated by digital platforms, amplifying attention towards their privacy and causing a decline in actual privacy disclosure actions [43,44].

The usefulness of recommendation information and the intrusiveness related to one’s privacy can trigger attitudinal ambivalence in users, on one hand, usefulness of recommendations increases users’ perception of functionality and value for products and services, while on the other, intrusiveness heightens the perceived risk of privacy leakage, leading to users having simultaneous positive and negative evaluations, thereby increasing the ambivalence of their attitudes. According to the APE model, individuals form both positive and negative evaluations based on automatic affective responses when stimulated externally, then through propositional processing, they employ reasoning to assess whether this automatic affective response is reasonable and whether this judgement aligns with their existing cognitive beliefs, inconsistency would lead to them applying cognitive effort to determine the veracity and the validity [26]. Specifically, high usefulness of recommendations elicits an automatic positive affective reaction in users; Meanwhile, recommendation intrusiveness, automatically triggers a negative affective reaction because it enhances perception of the risks associated with privacy breaches, such immediate assembly of positive and negative affects constructs attitudinal ambivalence in users leading to a sense of discrepancy, which requires the expenditure of significant cognitive resources to balance this psychological discomfort. Due to the cognitive limitations that individuals with high attitudinal ambivalence experience, during the process of information processing, they tend to actively reduce judgments that would increase cognitive load and are influenced by aspects such as informational framing, cue pointers, etc., to make decisions [23,25]. High recommendation usefulness combined with high informational intrusiveness can trigger high attitudinal ambivalence among users, making it more likely that they will expend fewer cognitive resources and make disclosure decisions based on the perceived benefits yielded by the high usefulness of the recommendations, leading to a congruity between instantly constructed attitudes and behavior. For low level recommendation intrusiveness scenarios, due to the reduced violations of privacy, users do not need to expend much cognitive resources, and the attitudes triggered by the perceived benefits and risk perception due to recommendation usefulness do not significantly change. Thus, the study proposes the following hypothesis:

H1: Recommendation usefulness and recommendation intrusiveness interact with user privacy disclosure.

H1a: Under conditions of high recommendation intrusiveness, compared to low recommendation usefulness, users have a higher tendency to disclose privacy with high recommendation usefulness.

H1b: Under conditions of low recommendation intrusiveness, recommendation usefulness does not significantly alter user’s privacy disclosure behaviors, yet it’s notably lower than the privacy disclosure rates under conditions of high information intrusiveness and high information usefulness.

Recommendation usefulness refers to a marketing strategy targeted at users, aiming to provide timely and precise recommendations for products and services that align with user needs based on their intentions [45]. Personalization can offer users a feeling of exclusivity and tailored experiences, boosting their perceived utility, augmenting their flow experience and immersion, elevating their willingness to use intelligent recommendation services, and thus encouraging privacy disclosure. Information aversion is defined as displeasure and aversion experienced by users when they perceive that personalized services in mobile commerce intrude upon their privacy. Information aversion then is the emotional response to what is perceived as the malicious invasion of privacy by online platforms and mobile devices, where such perceptions of personal privacy issues while using intelligent recommendation services generate negative emotions, leading users to engage in privacy avoidance behaviors. For example, if users feel that their location information is excessively tracked [46,47], the incessant push of nearby product information based on their geographical location can elicit dissatisfaction and aversion. Additionally, certain online platforms and mobile commerce services necessitate the provision of specific private information for functionality or software use, a demand which can cause users to react negatively.

Aside from the useful information provided by personalized recommendations that enhance users’ perception of functionality, the intrusiveness of recommendation services increases user unease regarding privacy violation. Users’ perception of functionality and feelings of dissatisfaction and aversion can enhance their cognitive and affective inconsistency. According to the APE model, even though information aversion can lead to negative affective responses in users, the positive cognitive cues from usefulness can elevate users’ perception of value, with users processing feelings of dissatisfaction and perceived value [48], they form cognitive and affective inconsistencies influencing decision-making. This cognitive and affective dissonance compels individuals to deploy additional cognitive resources to alleviate the discomfort, because individuals with cognitive and affective discordance are constrained by limited cognition in their information processing, they are predisposed toward judgments that reduce at the overload of their cognitive capacities. When perceived usefulness from recommendations is high, users trend toward expending fewer cognitive resources, utilizing the simple benefit judgment formed by positive cognitive clues of usefulness for disclosure decision-making, and are more likely to produce value perception propositions related to self through limited cognitive processing, overturning propositions induced by dissatisfaction and aversion from information aversion [48], and diminish internal resistance to behavioral intentions through instantly satisfying and functional benefits yielded by limited cognitive processing, thus positively promoting user privacy disclosure behavior. On this basis, the study proposes the following hypothesis:

H2: Compared to low recommendation usefulness and low information aversion, high recommendation usefulness and high information aversion contributes to higher tendencies toward privacy disclosure.

Privacy intrusiveness refers to the subjective feeling users have that digital businesses are using emerging technologies to excessively interfere with their private space, finding such recommendations intrusive [37]. Users are aware that digital enterprises collect private information excessively and beyond what is appropriate, often without the users’ knowledge and under the guise of privacy policies, such as disclosing location information for navigation services, yet digital platforms often track users’ behavioral patterns through high-frequency, unannounced sharing of user location data with third parties, infringing on privacy rights, leading to user concerns over privacy violations and driving them to adopt privacy avoidance behavior. Information interestingness refers to smart recommendation data presented in humorous, interesting ways that capture user interest, draw their attention, and elevate their affinity. Compared to dull information, users prefer to interact with humorous and entertaining content, which fosters positive emotions and mitigates the emergence of negative feelings, Eisend’ s study indicates that humorous and entertaining messages can heighten user attraction to advertising, thus generating positive effects [49], as fun and enjoyable content can bring pleasure and joy to users, thereby fulfilling their emotional needs, and it can also increase their fondness of products and services, positively impacting decision-making.

Privacy intrusiveness allows users to perceive digital enterprises’ intentions to manipulate personal information and a heightened perception of the threat to privacy leakage. Meanwhile, the information interestingness provides users with a sense of pleasure, increases the attractiveness of users, and meets their emotional needs. Privacy intrusiveness leads to perceptions of risk and threat among users, whereas the entertaining nature triggers positive emotions, thereby further accentuating cognitive and affective discrepancies. As individuals typically exhibit risk aversion, evidencing a psychological repulsion and avoidance to high risk, users with limited cognitive resources are preferred to intuit the extent of privacy intrusion, drawing on associative memory and reasoning to bolster their perception of privacy risk and threat. According to the APE model and theories of cognitive limitation, users generate negative emotions through automatic affective responses that result in psychologically ‘cut-your-losses’ judgments through limited cognition processing, which leads to a preference for risk averse strategies [50], and manifests as the aversion to risk that involves undertaking privacy avoiding behaviors [51]. Simultaneously, studies have found that the negative emotions arising from concerns over privacy leaks adversely affect the behavior of privacy disclosure [52]. Based on this, the study proposes the following hypothesis:

H3: Compared with conditions where privacy invasion is high and information interestingness is high, users are more likely to disclose privacy when both privacy invasion and interestingness are low.

### 3.2. ERP hypotheses

This research focuses on the P2 and N2 components as pivotal indicators, grounded in their distinct roles in emotional arousal and cognitive conflict resolution. These components align with the foundational principles of the limited cognitive resources theory and the APE (Appraisal-Prediction-Emotion) model. Specifically, the P2 component is typically intimately linked to attention allocation and emotional arousal. Drawing on the limited cognitive resources theory, when users perceive external information threats, their finite cognitive resources are prioritized for processing stages associated with threat assessment. The enhancement of P2 signifies the activation of these swift emotional reactions [53]. In conjunction with the APE model, P2 serves as a physiological indicator of emotional appraisal during the automatic processing phase. Conversely, the N2 component is primarily associated with conflict monitoring and cognitive control. When cognitive resources must address conflicts arising from internal and external information, competitive allocation of these resources intensifies, and N2 reflects the electrophysiological signatures of this process. According to the APE model, N2 can be utilized to characterize the dynamic cognitive conflict between emotional and cognitive processing [54]. Simultaneously, the P2 component indicates users’ emotional responses to privacy information, revealing the emotional conflict users face in privacy paradox situations, where they often feel ambivalent about privacy risks while enjoying convenience [55]. Conversely, the N2 component acts as a marker for understanding cognitive conflicts in users during the privacy decision-making process; variations in N2 can reflect the psychological conflicts encountered by users when dealing with privacy information [56], shedding light on the contradictions in their attitudes. Therefore, the selection of P2 and N2 components closely aligns with the research questions, providing theoretical and empirical support by revealing users’ emotional arousal (P2) and subsequent cognitive conflict (N2) during the privacy decision-making process, thereby offering a comprehensive portrayal of the neural mechanisms underlying users’ privacy behaviors.

The P2 component is primarily activated in the brain’s frontal and parieto-occipital regions, with a latency around 200ms, and can represent the level of risk perception, emotional valence, and degree of attentional bias. Research indicates that the P2 component has garnered widespread attention among scholars in studies of negative emotions. Ito has found that stimuli associated with negative emotions cause significant changes in P2 amplitude, with high negative emotions eliciting a larger P2 compared to lower negative emotions [53]. It’s also posited that stimuli inducing negative emotions produce larger P2 waves than those inducing positive emotions [57]. Furthermore, in preference selection tasks, the P2 amplitude is influenced by the degree of preference and congruity when consumers react to options they like or dislike [58]. According to the priority principle of cognitive resource allocation, when high-intrusiveness contexts of recommendation information trigger the threat assessment system (where privacy information is overly collected/shared), cognitive resources are primarily directed towards emotional processing. In this context, the mechanism of recommendation usefulness adheres to the emotional valence compensation model: high usefulness of recommendations can partially counteract threat perception and reduce negative emotions by offering immediate functional value (personalized services), thus suppressing the induced P2 amplitude; conversely, low usefulness leads to unaddressed negative emotions due to the absence of a compensatory mechanism, resulting in larger P2 amplitudes. This mechanism becomes ineffective in low-intrusiveness situations for recommendation information, as the threat assessment does not reach the activation threshold, leading resource allocation patterns to return to baseline levels. Additionally, according to the additive effect of emotional conflict, when high privacy intrusiveness (external threat) coexists with high information interestingness (internal attraction), a valence conflict emerges; this conflicting emotional experience enhances the P2 response through the collaborative activation of the amygdala-prefrontal cortex pathway. Based on this, the following hypotheses are proposed:

H4: There is a notable difference in individual P2 amplitude evoked by the usefulness of recommended information.

H4a: Under conditions of high recommendation intrusiveness, low recommendation usefulness is anticipated to evoke a higher P2 amplitude compared to high recommendation usefulness.

H4b: Under conditions of low recommendation intrusiveness, the usefulness of recommendation does not significantly affect P2 amplitude, but it is significantly larger than that induced by high information intrusiveness combined with high usefulness.

H5: Compared with low privacy intrusiveness and low information interestingness, high privacy intrusiveness and high information interestingness will induce higher P2 amplitude.

The N2 component is a peak negative component that emerges 200–350ms after stimulus presentation, typically distributed over the front, frontocentral midline, and central regions of the scalp. The N2 component originates from conscious cognitive processing and reflects cognitive conflict during individual decision-making. At the same time, the N2 component is indicative of task difficulty and individual cognitive effort. It has been discovered that decision risk can be characterized by the N2 component, reflecting the higher the decision risk, the more cognitive conflict it induces during decision-making [54]. Research shows that higher perceived risk in the decision-making process will lead to greater decision-making difficulty [59]. Therefore, uncontrollable threat cues can trigger greater cognitive conflict compared to controllable and severe threat clues, resulting in larger N2 waves [60]. Compared with the positive frame, the negative frame will cause consumers to have greater decision-making difficulties and cognitive conflicts, which will induce greater N2 amplitude [61].

According to the theory of limited cognition, individuals expend a certain amount of cognitive resources upon completing each task; however, cognitive resources are finite. When engaging in multiple tasks at the same time, cognitive resources need to be shared among these varied tasks. In particular, high usefulness of recommendations combined with high information intrusiveness triggers significant attitude ambivalence in users, resulting in a sense of dissonance that necessitates considerable cognitive resources to alleviate this psychological discomfort. Accordingly, in contexts of high recommendation information intrusiveness, the perceived benefits associated with low information usefulness fail to effectively mitigate the risk perception of high privacy intrusiveness when compared to high usefulness, resulting in increased cognitive conflict among individuals. Accordingly, in contexts of high recommendation information intrusiveness, the perceived benefits associated with low information usefulness fail to effectively mitigate the risk perception of high privacy intrusiveness when compared to high usefulness, resulting in increased cognitive conflict among individuals. During this time, the conflict signals in the anterior cingulate cortex become more pronounced, manifesting as an increased N2 amplitude. Additionally, high information aversion combined with high recommendation usefulness may create inconsistencies in users’ cognitive and emotional responses, further exacerbating psychological discomfort and necessitating substantial cognitive resources to restore balance. As a result, they tend to focus their limited cognitive resources on processing positive cognitive signals. When a goal conflict arises between recommendation usefulness and information aversion (functional utility vs. emotional rejection), additional prefrontal resources are needed for cognitive restructuring, with this regulatory process directly reflected in variations in N2 amplitude. Conversely, in situations of low information aversion and low recommendation usefulness, individuals possess more cognitive resources for privacy decision judgments, which in turn elicits larger N2 amplitudes.

Additionally, privacy intrusiveness causes users to recognize the threat of privacy violations, thereby increasing their risk perception. While information interestingness may elicit positive evaluations from users, the emotional needs arising from it fail to adequately mitigate users’ risk perception concerning privacy violations. This results in cognitive dissonance; the greater the sense of dissonance, the more cognitive resources individuals must invest to alleviate feelings of conflict and discomfort. In essence, high privacy intrusiveness triggers a threat-avoidance inclination, whereas high information interestingness stimulates a pleasure-seeking inclination; the competition between these conflicting motivations intensifies the activation of the dorsal anterior cingulate cortex, thereby amplifying the N2 response via the thalamocortical pathway. Consequently, the study puts forth the following hypotheses:

H6: There is significant variability in the N2 amplitude induced in individuals by the usefulness of recommendation information.

H6a: In the context of high recommendation intrusiveness, low recommendation usefulness induces a greater N2 amplitude compared to high recommendation usefulness.

H6b: Under low recommendation intrusiveness, there is no significant difference in the N2 amplitude due to recommendation usefulness, but it is notably larger than the N2 amplitude induced under conditions of high information intrusiveness combined with high recommendation usefulness.

H7: Lower recommendation usefulness combined with lower informational aversiveness induces a greater N2 amplitude compared to higher recommendation usefulness and informational aversiveness.

H8: A higher privacy intrusiveness paired with higher information interestingness results in a higher N2 amplitude, in comparison to low privacy intrusiveness and interestingness level.

**Fig 1:**
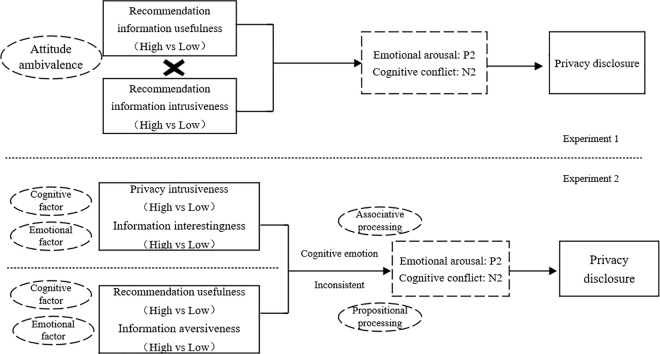
Research model.

Fig 1 presents the theoretical model of this research.

## 4. Methods

This research unfolds around the pivotal question of the impact of user attitudinal ambivalence due to the usefulness and intrusiveness of recommendation information on privacy decision-making mechanisms, and delves into whether users are more likely to make behavioral decisions based on cognitive or emotional factors when these structures are misaligned within the ambivalence. Through two EEG experiments, the study investigates the internal mechanisms by which user attitude ambivalence in smart recommendation contexts influences privacy disclosure behaviors. and the intrinsic cognitive processing of the individual is as follows:

Experiment 1 utilizes a 2 (recommendation information intrusiveness: high vs. low) * 2 (recommendation information usefulness: high vs. low) factorial design, aiming to investigate the mechanism by which user attitudinal ambivalence influences behavior in privacy disclosure, through ERP components associated with emotional arousal and cognitive conflict (namely P2 and N2 components), that serve to substantiate the impact dual attitudes have on the user’s privacy behavioral decision-making process, thereby laying the groundwork for Experiment 2. The study was conducted in accordance with the Declaration of Helsinki, and approved by Ethics Committee of Huaqiao University (M2023009 2023.4.19).

Building upon Experiment 1, Experiment 2 categorizes recommendation intrusiveness and usefulness according to cognitive and affective factors, resulting in divisions such as privacy intrusiveness (cognitive) and information interestingness (affective), as well as recommendation usefulness (cognitive) and information aversiveness (affective), The experiment is divided into two phases, primarily investigating the intrinsic mechanism of how inconsistencies between positive/negative cognition and positive/negative affect influence user behavioral decision-making, validating the impact on behavior decision-making by an individual’s immediate and genuine attitude formed through limited cognitive resources. The study was conducted in accordance with the Declaration of Helsinki, and approved by Ethics Committee of Huaqiao University (M2023009 2023.4.19).

### 4.1. Experiment 1

#### 4.1.1. Experimental subjects.

Experiment 1 uses a (recommendation information intrusiveness: high vs. low) *2 (recommendation information usefulness: high vs. low) mixed design. According to the standards posited in Cohen’s study, the sample size for the research was calculated using G*power 3.1, requiring a total sample of no less than 24 individuals [62]. The experiment recruited a total of 45 participants, all of whom were current university students, averaging around 24.5 years of age, right-handed, with no history of psychiatric illness, and either normal uncorrected or corrected vision. Before the experiment, the principal investigators explained the precautions to the subjects and obtained informed written; upon completion of the experiment, subjects were compensated based on the duration of their participation. During the experiment, the data from 2 subjects were deemed unusable due to poor condition, 1 participant was excluded owing to discomfort from contact lens use, leading to excessive artifacts in the data due to frequent blinking, with their effective Mark falling below 50%, the final effective number of subjects amounted to 42 (20 males and 22 females), which met the requirements for the sample size. This experiment is conducted from October to December 2023.

#### 4.1.2. Experimental materials.

Taking into account that participants might be affected by their familiarity with and the popularity of specific types of Apps, the research utilizes unbiased wording to name the Apps, such as “Software A,” for instance. According to the delineation of sensitive consumer information within the “Personal Information Protection Law,” this research selects 40 pieces of information as material. Ultimately, 15 types of information such as GPS, fingerprint, facial recognition, geographical location, and microphone were determined to comprise information with high privacy intrusion, with information types such as gender, age, ethnicity, weight, and educational background among the 15 selected as low privacy intrusion information. The choosing of recommended information usefulness mimics real Apps’ corresponding service functions, for high recommendation usefulness, the script reads, “If you allow ‘Software A’ to collect your information, it will accurately push notifications to you or give you access anytime, anywhere to...,” whereas, for low recommendation usefulness, it states, “If you allow ‘Software A’ to collect your information, it will push notifications to you or you will receive...” For instance, under equal conditions of high recommendation intrusiveness, a script such as ‘Allow “Software A” to collect your movement patterns to precisely recommend you nearby attractions and cuisine’ qualifies as materials with high informational usefulness; “Allow ‘Software A’ to collect your movement patterns to push notifications about attractions and cuisine,” etc., are considered to be low information usefulness materials. Before the official experiment, 80 non-participants were randomly invited to rate the level of intrusiveness and usefulness of the materials proposed by the experimental recommendations, t-tests on recommendation intrusiveness (158) =−11.89, p < 0.05, t-tests on the usefulness of information recommended (158) =−10.35, p < 0.05.

#### 4.1.3. Experimental procedure.

**Fig 2:**
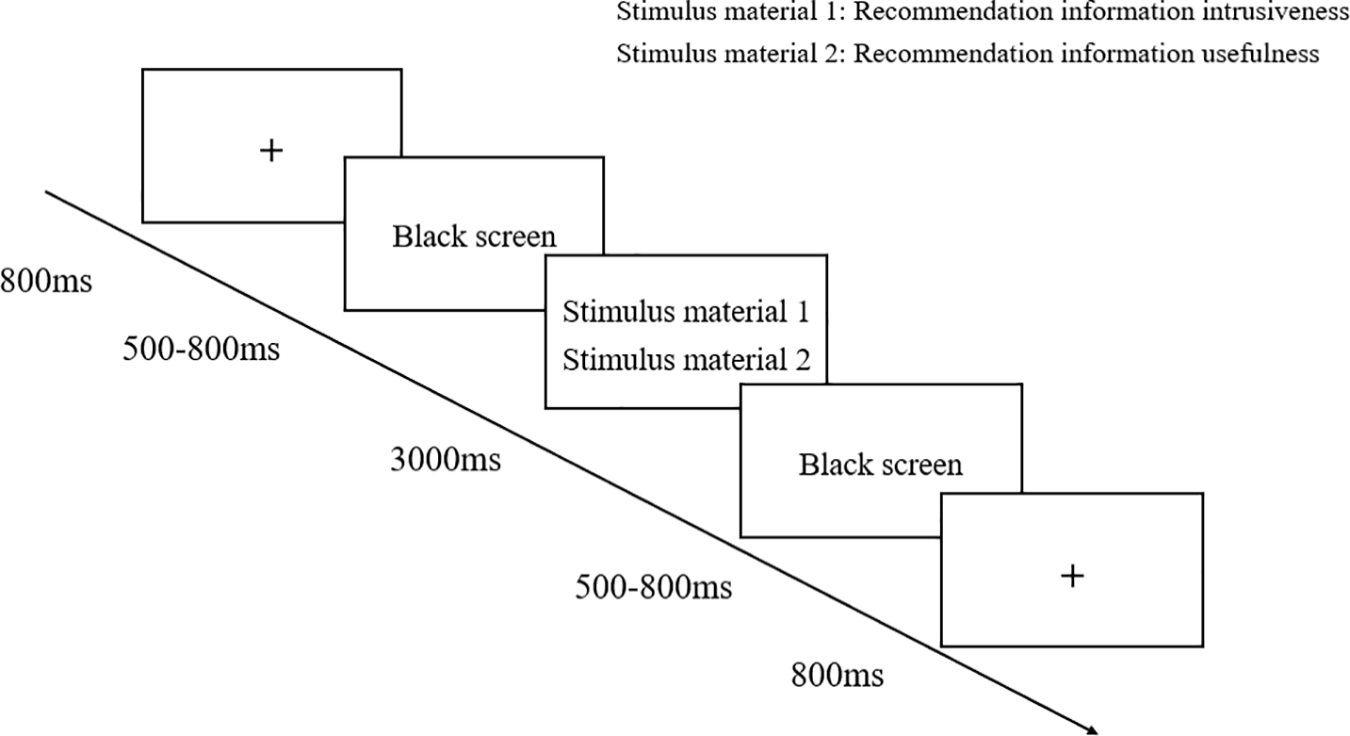
Experimental flowchart.

The experiment utilized an extended oddball paradigm, presenting the stimuli in equal proportion. Prior to the experiment, subjects were required to carefully read the experimental instructions, which the experimenter then explained to ensure they were correctly understood for optimal completion of the experiment. During the experiment, the screen randomly displayed stimuli with varying levels of recommendation intrusiveness and usefulness. The experiment consisted of two stages: practice and main session, with eight practice trials; following practice, the official experiment began with a press of the Q key. Initially, a “crosshair” (+) fixation point was displayed in the center of the computer screen for 500–800ms, succeeded by a blank screen phase of 800–1200ms, before the random appearance of one of four stimulus types, each sentence was displayed for 5000ms, and after an ensuing inter-stimulus interval of 800–1200ms, the subsequent trial commenced, subjects were required to respond with key presses based on the presented levels of recommendation intrusiveness and usefulness, where pressing the “F” key signified “refusal,” or disagreement, and the “J” key indicated “permission,” or agreement. The main experiment comprised two blocks, with each block including 88 trials, each participant engaged with 88 behavioral decision-making instances (preceded by a practice phase entailing eight trials), to remove sequential effects, each trial randomized the presentation of various stimuli materials. Subjects were instructed to concentrate throughout the experiment and to reduce behaviors like excessive blinking, swallowing, and significant movements, a 5–10minute rest break between the two blocks ensured that participants could continue the experiment with high quality, and the experimental procedure is shown in Fig 2.

### 4.2. Experiment 2

#### 4.2.1. Experimental subjects.

Experiment 2 has two stages and is primarily aimed at examining the impact of incongruity between positive/negative cognition and positive/negative affect on individuals’ privacy disclosure behavior. The study mainly focuses on Phase 1 with designs for high information aversiveness – high recommendation usefulness and low information aversiveness – low recommendation usefulness, and Phase 2 with designs for high information interestingness – high privacy intrusiveness and low information interestingness – low privacy intrusiveness. Following the standards recommended by Cohen, the sample size for the study was calculated using G*power 3.1 [59]. A minimum of 24 participants were required for the sample size. A total of 46 subjects were enrolled for the experiment, all of them being college students with an average age of approximately 23.5 years, right-handed, without psychiatric conditions, and having either normal vision or vision that is correctable to normal. Before the experiment, the principal investigators explained the precautions to the subjects and obtained informed written; upon completion of the experiment, subjects were compensated based on the duration of their participation. During the experiment, data from 6 individuals were rendered unusable due to poor condition during the procedure, and as their valid responses (Mark) were below 30%, they were excluded, resulting in a final tally of 42 valid subjects (22 males and 20 females), compliant with the sample size requisites. This experiment is conducted from October to December 2023.

#### 4.2.2. Experimental materials.

Considering the potential influence of familiarity with and popularity of certain types of apps among the participants, this study employed neutral terminology to name the apps, such as “PP Browser,” “T Show,” and “M Music,” to bring these into the experimental context. The materials for recommended usefulness and privacy intrusiveness were selected related to those used in Experiment 1. Information aversiveness was presented in an unwelcoming manner, where high information aversiveness contained statements such as “If you do not allow, you will not be able to use this software, and it will force-close,” “If permission is not granted, the use of this software will not be possible, and it will crash,” etc. Low information aversiveness was represented by phrases like “Without permission, you will be unable to use the above-mentioned feature,” etc. Information interestingness was manipulated through the use of a humorous and engaging delivery, with high informational entertainment materials involving subjects’ interests with a playful and humorous tone. Examples include “Turn on location services, be in-the-know with news hotspots right away,” “Activate GPS, become a dining expert in a second,” “Beautify your pictures with T Show, the more you showcase, the more spectacular it gets,” etc., while low informational entertainment was portrayed as “Permission will allow us to bring you information about local hotspots you might find interesting,” etc. Before the formal experiment, four doctoral students were invited to select the experimental stimuli materials, and 50 university students were invited to conduct subjective scoring of these materials. With t-values for information aversiveness (98) =−12.39, p < 0.05, and for informational entertainment (98) =−11.56, p < 0.05, the effectiveness of the experimental manipulation was assured.

#### 4.2.3. Experimental procedure.

The experiment utilizes an extension of the oddball paradigm with stimulus materials presented in equal proportions. Both Phase One and Phase Two of the experiment are conducted by different participants. Participants are asked to read the experimental instructions carefully before the experiment begins, and the examiner provides further explanation to ensure that the participants fully understand the process to perform the experimental tasks effectively. Taking Phase One as an example, during the experiment, different personalized cues and information interestingness stimuli are randomly presented in the middle of the screen. The experiment includes both practice and formal stages, beginning with eight practice trials. After these trials, the official experiment starts at the press of the Q key. Initially, a “+” fixation cross is displayed in the center of the computer screen for 500–800ms. Following a blank screen displayed for 800–1200ms, experiment stimulus sentences appear for 5000ms. Then, after another 800–1200ms blank screen, the next trial begins. Participants are required to press keys in reaction to different personalized cues and information interestingness propositions, where pressing “F” means “refuse” or to disagree, and pressing “J” means “permit” or to agree. There are 88 trials in total in the formal experiment phase, and each subject makes decisions on 88 behavioral actions (including the 8 practice trials). To eliminate sequence effects, each trial randomly presents different stimulus materials. The procedure for the experiment is the same as in Experiment 1.

### 4.3. Data extraction

In the experiment, EEG data were collected using the Neuroscan Synamp 2 Amplifier (Curry 7, Neurosoft Labs, Inc.), which has 64 electrodes with relative distances between scalp electrode points of 10% and 20%. The sampling rate was 1000 Hz, and all electrode impedances were kept below 10 kΩ. The FCZ electrode served as the online reference, and EEG data were processed with the EEGLAB 2023 plugin. EEG data for each participant were loaded into EEGLAB to verify channel positions. EEG data for each participant were loaded into EEGLAB to verify channel positions. Subsequently, the sampling rate was reduced to 500 Hz. The filter settings ranged from 0.1 Hz to 40 Hz, employing a band-pass filter between 48 Hz and 52 Hz to eliminate power line noise, while independent component analysis was utilized to remove artifacts associated with blinking, eye movements, and muscle activity. For ERP analysis, data from −200 ms to 800 ms prior to stimulus presentation were selected and baseline correction was conducted. The EEG components and time windows selected in this study included P2: 180–260 ms and N2: 260–320 ms, with analysis performed on electrode sites PZ, P4, PO3, POZ, PO4, FZ, FCZ, C3, CZ, and C4. Greenhouse-Geisser correction was implemented, and partial η^2^ effect sizes were reported.

## 5. Results

### 5.1. Behavioural results

In Experiment 1, for behavioral data, independent sample t-tests were conducted on the privacy disclosure rates of subjects in different recommendation information usefulness groups. The results showed that the privacy disclosure rate of the subjects in the low recommendation information usefulness group (M = 0.388, SD = 0.187) was significantly lower than that of the subjects in the high recommendation information usefulness group (M = 0.557, SD = 0.175), t(82)= −4.277, p = .000 < .001, indicating successful manipulation of the recommendation information usefulness material. ANOVA results demonstrated a significant main effect of recommendation information usefulness, F(1, 40)= 5.782, p = .021 < .05. As shown in Table 1. The main effect of recommendation information intrusiveness was not significant, while the interaction effect between recommendation information usefulness and recommendation information intrusiveness was significant, F(1, 40)=17.906, p = .000 < .001.

**Table 1:** ANOVA analysis of the intrusion and usefulness of recommended information on the privacy disclosure rate.

	*df*	*F*	*Sig.*	*η* ^*2*^
Recommendation information usefulness	1	5.782	0.021	0.342
Recommendation information intrusiveness	1	0.425	0.520	0.015
Recommendation information usefulness * intrusiveness	1	17.906	0.000	0.482

In the group with high recommendation information intrusiveness, the privacy disclosure rate of the subjects in the high recommendation information usefulness group (M = 0.579, SD = 0.038) was significantly higher than that of the subjects in the low information usefulness group (M = 0.354, SD = 0.041), F(1, 19)=18.779, p = .000 < .001. In the group with low recommendation information intrusiveness, there was no significant difference between the privacy disclosure rate in the high usefulness group (M = 0.536, SD = 0.038) and the low usefulness group (M = 0.450, SD = 0.041), F(1, 19)=2.725, p = .107 > .05. As shown in Table 2. Additionally, under the condition of high intrusiveness and high usefulness of recommended information, the privacy disclosure rate (M = 0.579, SD = 0.175) was significantly higher than that of subjects in the low intrusiveness and low usefulness group (M = 0.450, SD = 0.193), F(1, 19)=10.385, p = .004 < .01. From the results mentioned above, we can infer that recommendation information usefulness has a significant effect on privacy disclosure. Specifically, under conditions of high intrusiveness, subjects are more inclined to disclose their privacy in the state of high usefulness compared to low usefulness, while under low intrusiveness, recommendation information usefulness does not significantly impact the privacy disclosure rate, which was significantly lower than under the condition of high intrusiveness and high usefulness. Therefore, hypotheses H1, H1a, and H1b are supported.

**Table 2:** Simple effect analysis of intrusiveness and usefulness of recommended information on privacy disclosure rate.

Recommendation information intrusiveness	Recommendation information usefulness	M	SD	*F*	*Sig.*
High recommendation information intrusiveness	High usefulness	0.579	0.038	18.779	0.000
	Low usefulness	0.353	0.041		
Low recommendation information intrusiveness	High usefulness	0.536	0.038	2.279	0.107
	Low usefulness	0.450	0.041		

Within the behavioral data of Experiment 2, the analysis of variance demonstrated that the privacy disclosure rate for participants in the low recommendation usefulness-low information averseness group was significantly lower (M = 0.511, SD = 0.252) than for those in the high recommendation usefulness-high information averseness group (M = 0.796, SD = 0.200), F(1, 19)= 16.273, p = .001 < .01. Furthermore, participants in the low privacy intrusiveness-low information interestingness group had a significantly higher rate of privacy disclosure (M = 0.518, SD = 0.053) compared to those in the high privacy intrusiveness-high information interestingness group (M = 0.220, SD = 0.054), F(1, 19)=17.679, p = .000 < .001. As shown in Table 3.

**Table 3:** The variance analysis of recommendation usefulness – information averseness and privacy intrusiveness – information interestingness on privacy disclosure rate.

	M	SD	*F*	*Sig.*
Low recommendation usefulness-low information averseness	0.511	0.252	16.273	0.001
High recommendation usefulness- high information averseness	0.796	0.200		
Low privacy intrusiveness-low information interestingness	0.518	0.053	17.679	0.000
High privacy intrusiveness- high information interestingness	0.220	0.054		

From the above results, it can be understood that, compared to the low recommendation usefulness and low information averseness conditions, subjects showed a greater disposition towards privacy disclosure under the conditions of high recommendation usefulness and high information averseness. Similarly, compared to conditions of high privacy intrusiveness and high information interestingness, subjects were more inclined to disclose private information under conditions of low privacy intrusiveness and low information interestingness, thus confirming hypotheses H2 and H3.

### 5.2. EEG results

#### 5.2.1. P2 component results.

Based on prior research conducted by scholars on the P2 and N2 components, the selection of electrode sites for the P2 component includes PZ, P4, PO3, POZ, PO4, with an analysis of the 180–260ms time window. For the N2 component, the electrode sites chosen are FZ, FCZ, C3, CZ, C4, with the analysis focusing on the 260–320ms time window.

Based on the multivariate test results of Experiment 1, the main effect of recommended information intrusiveness is marginally significant, F(1,40)=15.499, p = .000 > .050. The main effect of recommended information usefulness is significant, F(1,40)=15.499, p = .000 < .001. The P2 amplitude for low recommendation information usefulness (M = 6.583, SD = 0.354) is significantly greater than that for high recommendation information usefulness (M = 4.715, SD = 0.380). Therefore, hypothesis H4 is supported. The main effect for electrode sites is significant, F(4,37)= 24.367, p = .000 < .001. The interaction between recommendation information intrusiveness and recommendation information usefulness is significant, F(1,40)= 25.435, p = .000 < .001. As shown in Table 4.

**Table 4:** Multivariate test analysis of P2 components in experiment 1.

	*df*	*F*	*Sig.*	*η* ^*2*^
Recommendation information usefulness	1	15.499	0.000	0.379
Recommendation information intrusiveness	1	2.851	0.099	0.050
P2	9	24.367	0.000	0.542
Recommendation information usefulness * intrusiveness	1	25.435	0.000	0.725

In the group with high recommendation information intrusiveness, the usefulness of recommended information significantly affects the amplitude of the P2 component, F(1, 19)=6.928, p = 0.012 < .05. The P2 amplitude evoked in subjects with low usefulness of recommendation information (M = 6.938, SD = .501) is significantly larger than that evoked in subjects with high recommendation information usefulness (M = 4.259, SD = .538). As shown in Table 5.

**Table 5:** Analysis of simple effects of high intrusion and usefulness of recommendation information on P2 components.

Recommendation information intrusiveness	Recommendation information usefulness	M	SD	df	*F*	*Sig.*
High recommendation information intrusiveness	High usefulness	5.171	0. 538	19	6.928	0.012
	Low usefulness	6.938	0. 501	19		

**Fig 3:**
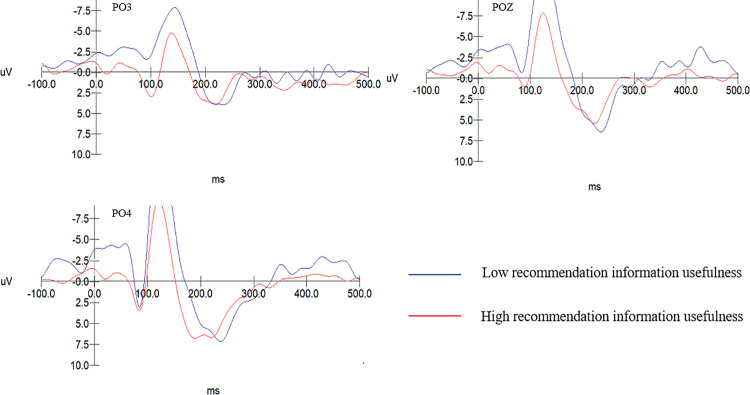
The P2 amplitude associated with different levels of recommendation information usefulness within the high recommendation information intrusiveness group.

**Fig 4:**
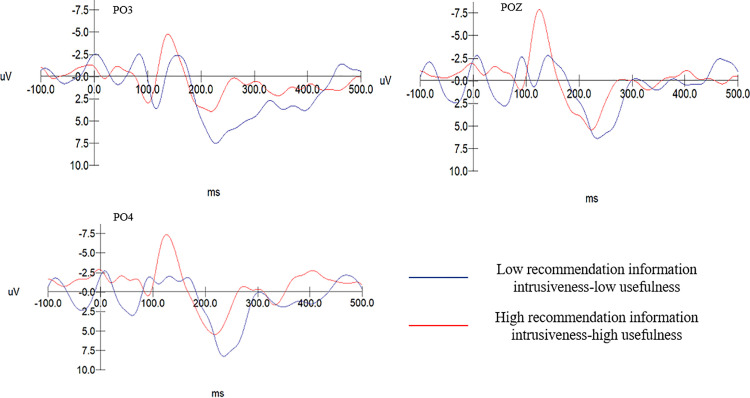
P2 amplitudes in the low recommendation information intrusiveness-low usefulness group compared with the high recommendation information intrusiveness-high usefulness group.

In the group with low recommendation information intrusiveness, the usefulness of recommendation information does not have a significant effect on the amplitude of the P2 component, F(1, 19)= 2.869, p = 0.098 > 0.05. Differences in the amplitude of the P2 component across varying levels of recommended information usefulness within the high informational intrusiveness group can be specifically seen in Fig 3. The P2 amplitude evoked in the low recommendation intrusiveness-low recommendation information usefulness group (M = 6.229, SD = .442) is significantly greater than that in the high intrusiveness-high usefulness group (M = 5.171, SD = .442), F(1, 19)= 6.228, p = 0.021 < .05. Consequently, hypotheses H4a and H4b are upheld. For detailed information, as detailed in Fig 4.

**Fig 5:**
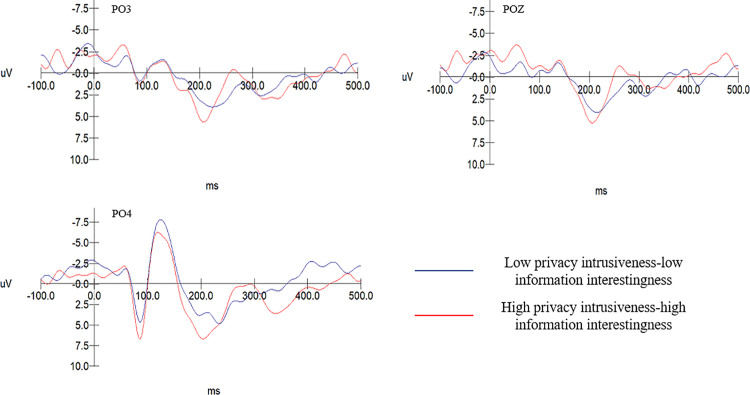
P2 amplitude comparisons between the low privacy intrusiveness-low information interestingness group and the high privacy intrusiveness-high information interestingness group.

The EEG results from Experiment 2 indicate that the P2 amplitude induced in the low privacy intrusiveness-low information interestingness group (M = 5.300, SD = .668) was significantly lower than in the high privacy intrusiveness-high information interestingness group (M = 9.534, SD = .668), F(1,19)=20.075, p = .000 < .001, as specifically shown in Fig 5.

#### 5.2.2. N2 component results.

The multivariate test results from Experiment 1 indicate a nonsignificant main effect of recommendation information intrusiveness, F(1, 40)=1.805, p = 1.187 > .050. The main effect of electrode sites is significant, F(4, 37)=40.632, p = .000 < .001, η^2^ = 0.741. The interaction between recommendation information intrusiveness and usefulness is significant, F(4, 37)=3.277, p = .021 < .05, η^2^ = 0.349.

**Fig 6:**
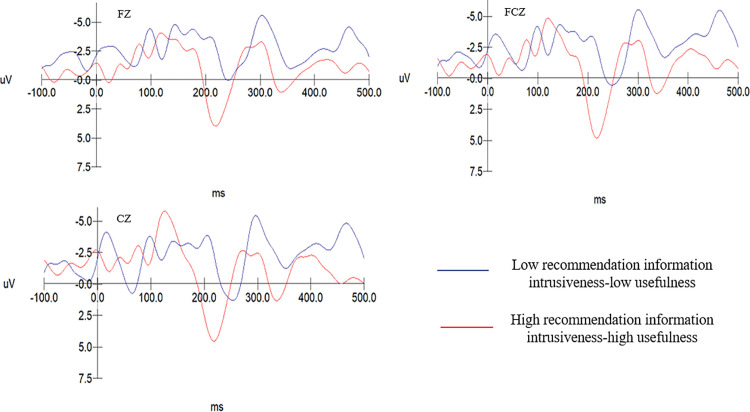
N2 wave amplitude across different levels of recommendation information usefulness in the high recommendation intrusiveness group.

In the group with high recommendation information intrusiveness, the usefulness of recommendation information has a significant main effect on the N2 component, F(1, 40)=15.291, p < .001. The N2 amplitude evoked in subjects with low usefulness of recommendation information (M = −4.269, SD = .508) is significantly greater than that evoked with high recommendation information usefulness (M = −1.928, SD = .481), F(1, 19)=7.575, p = .009 < .05. As shown in Table 6. In the low recommendation information intrusiveness group, the usefulness of recommendation information has no significant effect on the amplitude of the N2 component, F(1, 19)=2.154, p = 0.08 > .05). The N2 amplitude across different levels of recommendation information usefulness in the high intrusiveness group is shown in Fig 6. The N2 amplitude elicited in the low recommendation intrusiveness-low usefulness group (M = −4.921, SD = .438) is significantly greater than that elicited in the high intrusiveness-high usefulness group (M = −1.918, SD = .438), F(1, 19)=24.475, p = 0.000 < .001. Therefore, H6, H6a, and H6b are accepted.

**Table 6:** Analysis of simple effects of high intrusion and usefulness of recommendation information on N2 components.

Recommendation information intrusiveness	Recommendation information usefulness	M	SD	df	*F*	*Sig.*
High recommendation information intrusiveness	High usefulness	−1.928	0.481	19	7.575	0.009
	Low usefulness	−4.269	0. 508	19		

**Fig 7:**
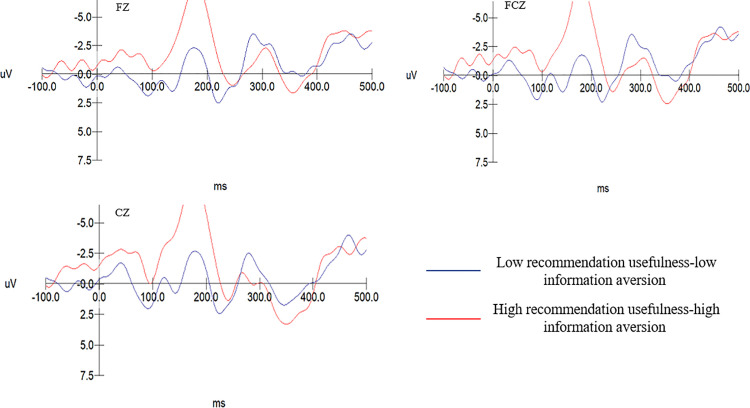
N2 amplitude between the low usefulness-low aversion and the high usefulness-high aversion group.

**Fig 8:**
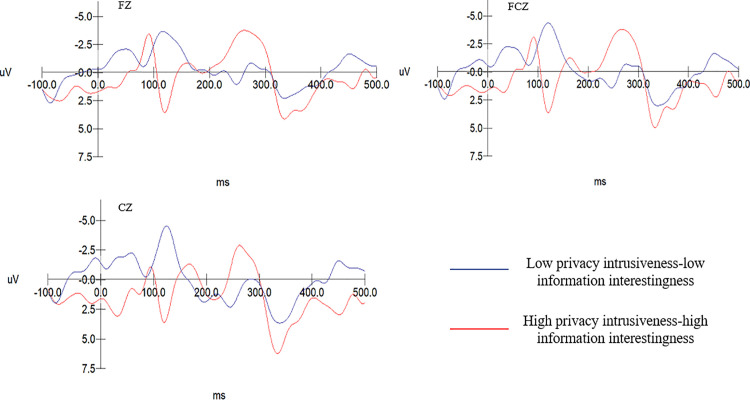
N2 amplitude comparison between the low privacy intrusiveness-low entertainment group and the high privacy intrusiveness-high entertainment group.

The EEG data results from Experiment 2 show: The N2 amplitude evoked in the low recommendation usefulness-low information aversion group (M = −4.056, SD = .437) is significantly greater than that in the high recommendation usefulness-high information aversion group (M = −0.705, SD = .437), F(1, 20)= 29.414, p = .000 < .001. For the group with low privacy intrusiveness and low information interestingness, the N2 amplitude (M = −0.437, SD = .474) is notably less than that for the group with high privacy intrusiveness and high entertainment (M = −3.768, SD = .474), F(1, 20)= 24.748, p = .000 < .001. As shown in Table 7. Consequently, hypotheses H7 and H8 are confirmed. Specific details are shown in Figs 7 and 8.

**Table 7:** The variance analysis of recommendation usefulness – information averseness and privacy intrusiveness – information interestingness on N2 components.

	M	SD	*F*	*Sig.*
Low recommendation usefulness-low information averseness	−4.056	0.437	29.414	0.000
High recommendation usefulness- high information averseness	−0.705	0.437		
Low privacy intrusiveness-low information interestingness	−0.437	0.474	24.748	0.000
High privacy intrusiveness- high information interestingness	−3.768	0.474		

## 6. Discussion

### 6.1. Conclusions

This study, utilizing two EEG (electroencephalograph) experiments and focusing on attitude instability as its subject matter, investigated the internal cognitive mechanisms of user attitudinal ambivalence with respect to privacy disclosure. It further substantiated how discrepancies in individual cognition and emotion can affect decision-making behavior. The study incorporated the usefulness of recommendation information and the intrusiveness of recommendation information as variables of attitudinal ambivalence. Additionally, it divided the usefulness of recommendation information into recommendation usefulness and information interestingness through cognitive and affective factors; likewise, the intrusiveness of recommendation information was divided into privacy intrusiveness and information aversion. Consequently, the study probed into how inherent instabilities within a user’s attitude system influence behavioral decision-making. By conducting EEG experiments, the study analyzed brain components such as P2 and N2, and button-press responses—to reflect the real and specific cognitive processes underlying attitudes towards privacy disclosure manifested through behavior. The research findings obtained from this study are as follows:

(1) Under conditions of high recommendation information intrusiveness, user privacy disclosure when recommendation information was considered to be of high usefulness was significantly greater than their privacy disclosure when recommendation usefulness was low. However, under conditions of low recommendation information intrusiveness, the usefulness of recommendation information did not have a significant differential effect on user privacy disclosure. Nevertheless, the privacy disclosure rate of users under conditions of both high recommendation information intrusiveness and usefulness was significantly higher than that under conditions of both low intrusiveness and low usefulness of recommendation information. This indicates that individuals with high attitudinal ambivalence are more inclined to disclose their privacy, laying the foundation for the results of Experiment 2.
(2) Users displayed a significantly lower rate of privacy disclosure when presented with clues indicating low usefulness of recommendations and low information aversion compared to when they were exposed to high recommendation usefulness and high information aversion, suggesting that individuals with high attitudinal ambivalence are more disposed to privacy disclosure. Moreover, when confronted with high privacy intrusiveness and high amusement information cues, individuals showed a significantly reduced rate of privacy disclosure compared to the rate under low privacy intrusiveness and low information interestingness conditions, implying that individuals with high attitudinal ambivalence tend to be less likely to disclose privacy.
(3) Under conditions of high recommendation information intrusiveness, the P2 and N2 wave amplitudes induced by high usefulness of recommendation information were significantly lower than those induced by low usefulness of recommendation information. Additionally, the P2 and N2 amplitudes elicited under conditions of low privacy intrusiveness and low information interestingness were significantly lower than those elicited under conditions of high privacy intrusiveness and high information interestingness. Furthermore, the P2 amplitudes elicited by high usefulness combined with high information aversiveness were significantly lower than those P2 amplitudes elicited under low recommendation usefulness and low information aversiveness.

The results of Experiment 1 are consistent with previous conclusions indicating that high intrusiveness of recommendations information coupled with high usefulness of recommendations induces high attitudinal ambivalence in individuals. This ambivalence elicits a sense of discomfort in users, necessitating the expenditure of considerable cognitive resources to mitigate this psychological unrest. Owing to the cognitive constraints affecting highly ambivalence individuals, there is a preference to actively reduce cognitive load during information processing. Consequently, users are likely to expend fewer cognitive resources and make disclosure decisions based on simple profit judgments driven by the usefulness of recommended information. This leads to the immediate construction of attitudes that are consistent with behavior.

Building on the results of Experiment 1 by subdividing cognitive and affective factors, it was discovered through a discrepancy between positive cognition and negative emotion that high recommendation usefulness coupled with high information aversiveness led individuals to experience attitude ambivalence. This ambivalence consumed considerable cognitive resources, which were alleviated by those individuals favoring behavior decisions based on the positive cues of recommendation usefulness thereby reducing cognitive expenditure. Conversely, through inconsistency between negative cognition and positive affect, it was discovered that when individuals experienced attitudinal ambivalence induced by high privacy intrusiveness combined with high information amusement, because of an aversion to losses and the instability of their own attitudes, they tended to base behavior decisions more on the negative cues associated with privacy intrusiveness.

### 6.2. Research contributions

This study makes the following theoretical contributions. Firstly, this research examines the influence of users’ attitudinal ambivalence on privacy disclosure behavior from the vantage point of attitude instability, thereby furnishing a novel explanatory mechanism for the study of privacy paradoxes. Traditional research typically relies on self-reported attitudes from users after careful deliberation [6,8]; however, this approach frequently fails to accurately mirror users’ privacy decisions during the dynamic information processing phase. The constraints of these methodologies have impeded a comprehensive exploration of the cognitive processes underlying privacy behaviors. Existing studies utilize techniques such as magnetic resonance imaging and event-related potentials to investigate privacy attitudes with greater precision and authenticity, surmounting previous measurement limitations and better aligning with the cognitive processing characteristics of users in real-world online decision-making contexts [9]. Nevertheless, these studies generally presume the stability of individual attitudes, neglecting the instability of attitudes during actual decision-making by users. Consequently, this study endeavors to investigate how attitude instability impacts users’ privacy disclosure behavior, analyzing the intrinsic mechanisms through which limited cognitive resources influence privacy decisions amidst cognitive and emotional dissonance. By focusing on the instability of user attitudes, this study elucidates the complexity of privacy disclosure behaviors and offers fresh perspectives for research on the privacy paradox.

Secondly, this study, grounded in the theory of bounded rationality and the APE model (Approach/Avoidance, Prevention/Promotion, and Engagement/Disengagement), has broadened their applicability within the privacy domain. Traditional research, typically anchored in theories such as privacy calculus theory or construal level theory [6,8], views attitudes like privacy concerns and privacy interests as deliberate, rationally processed, and self-reported evaluative representations. However, the actual decision-making processes of users concerning privacy often diverge from these deliberate self-reported outcomes and fail to account for the dynamic construction of attitudes. Furthermore, despite existing research employing neuroscientific techniques [13] and grounded in self-perception and attitude metacognitive theory to decipher the privacy paradox through a refined, more realistic cognitive processing framework [14,15], these accounts have overlooked the limitations of an individual’s cognitive resources. In genuine decision-making scenarios, users are influenced by attitudes shaped under the constraints of these limited cognitive resources. Nevertheless, limited research has explored the impact of such instantaneously formed privacy attitudes—rooted in bounded cognition related to benefits and risk assessment—on decision-making. Therefore, this research, leveraging the theory of limited cognitive resources and the APE model, along with ERPs technology, examines the cognitive processes underlying user attitudinal ambivalence in privacy disclosure and investigates the influence of mismatches between positive/negative cognition and positive/negative emotion, in conjunction with limited cognitive resources, on privacy disclosure decisions. This enhances the application of bounded rationality theory and the APE model in privacy paradox research.

Finally, this study utilizes ERPs technology to offer novel insights into experimental research pertaining to the cognitive mechanisms underlying user online behavior. Traditional research methodologies, such as interviews and behavioral assessments, enable comprehensive studies yet often fail to capture users’ online cognition and immediate behavioral responses in real time [9,15]. Consequently, this study analyzes brain electrical signals from a neurophysiological perspective to delve into the underlying mechanisms of users’ attitudinal contradictions concerning privacy disclosure, taking into account the constraints posed by limited cognitive resources, emotional arousal, and the processing of cognitive conflicts in real-time scenarios. By directly acquiring physiological electrical signals from the brain, this study precisely quantifies immediate cognitive and emotional responses, thereby mitigating the subjective biases inherent in traditional methodologies and making a preliminary foray into unveiling the “black box” of consumer cognitive processing. This endeavor lays the groundwork for subsequent research endeavors.

### 6.3. Practical significance

In the digital intelligence era, aggregating and analyzing data has emerged as a pivotal aspect concerning the application of advanced smart technologies [63]. In areas like e-commerce and social networking, iterative algorithms generate precise data for personalized marketing and innovative services. The success of this business model is underpinned by massive amounts of personal data, enabling the transformation of online user privacy information into data resources with potential consumer behavior, increasingly possessing rarity and economic value. How can big data companies more precisely interpret the online privacy preferences of users, and how might they incentivize users to disclose personal data while safeguarding their privacy rights? These inquiries will be tackled by offering concrete recommendations for the design of user experiences and the formulation of privacy policies, grounded in the research findings.

On the one hand, this study reveals that users exhibiting high levels of attitude ambivalence are more prone to disclosing their privacy under circumstances where the perceived usefulness of recommended information is high and its intrusiveness is also significant. Their decision-making processes are predominantly influenced by positive cues, such as the utility of the recommendations. This finding suggests that digital platforms ought to prioritize reducing attitude ambivalence and optimizing the presentation of information when devising user experiences. For example, in the design of recommended content, platforms should initially enhance the engagement and utility of the recommended information, while concurrently minimizing the intrusiveness of the pushed information to alleviate users’ perceptions of privacy risks. EEG findings indicate that users in situations of high attitude ambivalence expend considerable cognitive resources during their decision-making processes. Consequently, interface design should simplify decision pathways to alleviate the cognitive load on users during privacy disclosure decisions. Specifically, clear visual design, transparent informational frameworks [64], and sophisticated recommendation support tools can facilitate this objective, enabling users to swiftly comprehend the recommended content and its associated privacy implications, thereby enhancing user experience and mitigating decision-making stress. Furthermore, platforms can implement a tiered recommendation strategy, customized to accommodate diverse user types, dynamically adjusting the content and frequency of information to cater to users’ varied needs for recommendations.

On the other hand, this study discovers that the conjunction of high privacy intrusiveness and high information appeal markedly diminishes users’ inclination to disclose their privacy, mirroring users’ prudent conduct in response to discrepant cues of negative cognition (privacy intrusiveness) and positive affect (information engagement). Platforms ought to underscore the beneficial aspects of data utilization within their privacy policies, for example, by furnishing concrete exemplars of how data collection augments user experience (such as refining recommendation precision and personalization) [65], in order to bolster users’ positive perceptions. Concurrently, the privacy policy should unequivocally articulate the dedication to safeguarding users’ privacy, thereby mitigating users’ perceptions of privacy risks. EEG findings suggest that users in scenarios characterized by high attitude ambivalence allocate more cognitive resources to alleviate feelings of dissonance. This indicates that privacy policy design should endeavor to streamline intricate clauses, enhancing readability and transparency, to alleviate the cognitive load on users when interpreting the policies. For instance, through the utilization of visual aids (such as privacy infographics) or the presentation of policy terms in a phased manner, which facilitates users’ rapid comprehension of the privacy policy’s core components. Furthermore, considering users’ behavioral predispositions towards loss aversion, platforms can institute notification systems that provide prompt feedback whenever users’ privacy decisions may entail risks, aiding users in more adeptly weighing the pros and cons.

### 6.4. Limitations and future prospects

Although this research has certain theoretical relevance and practical implications, it is not without its limitations. First, this research emphasizes the effects of attitude instability on user decision-making behaviors. While the attitude system encompasses both stability and instability, existing studies have primarily examined the intrinsic cognitive mechanisms of instability in decision-making, limiting a holistic understanding of the complexity within the attitude system. Future studies should aim to investigate both the stability and instability of attitudes concurrently, examining their combined influence on privacy decisions across various information processing approaches. Adopting this integrated perspective allows for a deeper comprehension of the cognitive processes individuals undergo when confronted with privacy decisions, enriching research outcomes and strengthening the credibility of conclusions. This methodology not only aids in uncovering the dynamic features of attitude change but also offers theoretical backing for the development of privacy protection strategies. Second, this study’s sample primarily consists of college students. Although this group has significantly higher internet usage and online time in the digital age and is a major consumer of personalized recommendations, the homogeneity of the sample limits the broader applicability of the research. Although college students may have consistent intrinsic cognition regarding privacy information in online contexts, effectively reducing sampling error and improving external validity, the study still has certain limitations considering the homogeneity of the demographic structure, the relatively small sample size, and the influences of age, culture, and ideological backgrounds. Future research should consider including samples from different age groups, cultural backgrounds, and social demographics to enhance the generalizability and persuasiveness of the research conclusions. This will help to gain a more comprehensive understanding of the differences in privacy information cognition across various groups, thereby providing more targeted recommendations for the formulation of privacy protection policies. Third, this study is limited by the EEG experimental method, primarily focusing on the effects of information comprehensibility and consistency on consumers’ selective exposure, as well as the cognitive neural mechanisms of users’ attitude instability regarding privacy behaviors. However, this focus has led the study to overlook other potentially significant factors that may influence privacy behaviors, such as social environment, habitual patterns, and individuals’ risk preferences. Future research should comprehensively consider these variables, including age, education level, internet usage habits, and risk perception, to thoroughly analyze their potential impact on privacy decision-making. Future research should comprehensively consider these variables, including age, education level, internet usage habits, and risk perception, to thoroughly analyze their potential impact on privacy decision-making.
